# Cortical involvement in celiac disease before and after long-term gluten-free diet: A Transcranial Magnetic Stimulation study

**DOI:** 10.1371/journal.pone.0177560

**Published:** 2017-05-10

**Authors:** Manuela Pennisi, Giuseppe Lanza, Mariagiovanna Cantone, Riccardo Ricceri, Raffaele Ferri, Carmela Cinzia D’Agate, Giovanni Pennisi, Vincenzo Di Lazzaro, Rita Bella

**Affiliations:** 1 Spinal Unit, Emergency Hospital “*Cannizzaro*”, Catania, Italy; 2 Department of Neurology IC, I.R.C.C.S. “*Oasi Maria SS*.”, Troina, Enna, Italy; 3 Department of Medical and Surgical Sciences and Advanced Technologies, Section of Neurosciences, University of Catania, Catania, Italy; 4 Gastroenterology and Endoscopy Unit, University of Catania, Catania, Italy; 5 Department “*Specialità Medico-Chirurgiche*”, University of Catania, Catania, Italy; 6 Institute of Neurology, Campus Bio-Medico University, Rome, Italy; University of Ottawa, CANADA

## Abstract

**Objective:**

Transcranial Magnetic Stimulation in *de novo* patients with Celiac Disease previously revealed an imbalance in the excitability of cortical facilitatory and inhibitory circuits. After a median period of 16 months of gluten-free diet, a global increase of cortical excitability was reported, suggesting a glutamate-mediated compensation for disease progression. We have now evaluated cross-sectionally the changes of cortical excitability to TMS after a much longer gluten-free diet.

**Methods:**

Twenty patients on adequate gluten-free diet for a mean period of 8.35 years were enrolled and compared with 20 *de novo* patients and 20 healthy controls. Transcranial Magnetic Stimulation measures, recorded from the first dorsal interosseous muscle of the dominant hand, consisted of: resting motor threshold, cortical silent period, motor evoked potentials, central motor conduction time, mean short-latency intracortical inhibition and intracortical facilitation.

**Results:**

The cortical silent period was shorter in *de novo* patients, whereas in gluten-free diet participants it was similar to controls. The amplitude of motor responses was significantly smaller in all patients than in controls, regardless of the dietary regimen. Notwithstanding the diet, all patients exhibited a statistically significant decrease of mean short-latency intracortical inhibition and enhancement of intracortical facilitation with respect to controls; more intracortical facilitation in gluten-restricted compared to non-restricted patients was also observed. Neurological examination and celiac disease-related antibodies were negative.

**Conclusions:**

In this new investigation, the length of dietary regimen was able to modulate the electrocortical changes in celiac disease. Nevertheless, an intracortical synaptic dysfunction, mostly involving excitatory and inhibitory interneurons within the motor cortex, may persist. The clinical significance of subtle neurophysiological changes in celiac disease needs to be further investigated.

## Introduction

### Background and rationale

Celiac disease (CD) is an immune-mediated inflammatory disorder of the small intestine due to gluten sensitivity leading to alteration of the mucosal architecture and impairment in the absorption of nutrients [1]. The prevalence of CD in the general population is approximately 1% [2, 3]. Diagnosis is based on clinical suspicion and a subsequent confirmation by laboratory tests, duodenal biopsy and, in some cases, genetic testing. CD is now regarded as a complex systemic disorder with highly variable clinical presentation and frequent extraintestinal involvement [4]. Cerebellar ataxia, seizures, and peripheral neuropathy are the most common neurological manifestations of CD; they may either follow the appearance of the disease or be present at its onset. Furthermore, there may be also a silent neurological involvement during the course of the disease; the opposite case is also true, considering that pure central and/or peripheral nervous system diseases and gut histopathological changes can be seen in some CD patients without typical intestinal symptoms [4]. These scenarios highlight the importance of a diagnostic tool suitable for detecting even the silent presence or progression of the disease.

In this regard, Transcranial Magnetic Stimulation (TMS) has emerged as a non-invasive neurophysiological technique capable to probe the central motor conductivity and excitability of cortico-spinal and cortico-cortical circuits in the normal brain. as well as to Moreover, TMS is able to unveil and monitor motor system impairment in the pre-clinical phase of several neuropsychiatric disorders or systemic diseases with CNS involvement [5–12], also with potential therapeutic purposes [13, 14]. Finally, by evaluating the effects of agonists or antagonists for specific neurotransmitters, TMS can selectively and non-invasively explore the function of glutamatergic, gamma-aminobutyric-acid (GABA)-ergic, monoaminergic, and cholinergic central circuits (the so called “pharmaco-TMS”) [15, 16].

In a previous study aiming to evaluate the effect of the gluten-mediated immune disorder on cerebral cortex function [17], we investigated the profile of cortical excitability to TMS of 20 neurologically asymptomatic *de novo* CD patients. The protocol included single- and paired-pulse TMS-derived measures of motor cortex and cortico-spinal excitation (resting motor threshold—rMT; motor evoked potentials—MEPs), inhibition (cortical silent period—CSP; short-latency intracortical inhibition—SICI), and facilitation (intracortical facilitation—ICF). Compared to healthy controls, a statistically significant reduced ICI (0.3 *vs*. 0.2, *p* <0.045) and enhanced ICF (1.1 *vs*. 0.7, *p* <0.042) were observed. Based on these findings, the authors suggested the presence of subclinical functional changes of GABAergic and glutamatergic neurotransmission in CD [17]. When the same cohort was re-evaluated after a relatively short period of gluten-free diet (GFD) (median of 16 months), it was observed that the improvement of gastrointestinal symptoms was not paralleled by a normalization of cortical excitability but, unexpectedly, by a further increase of cortical excitability [18]. We speculated that this finding might represent a compensatory phenomenon triggered by gluten exposure that, for unknown reasons, persist after the beginning of GFD. Alternatively, the length of the follow-up period or the adherence to the diet could have been insufficient for a complete recovery [18].

It remains unknown how GFD affects subclinical neurological abnormalities. However, there is information about the neurophysiopathology of CD after alimentary therapy In this context, the efficacy of a prolonged diet was reported in CD-associated epilepsy [19]. More interestingly, a recent EEG investigation found subclinical abnormalities in 48% of children, which disappeared in 78% of them (namely, 48% of children with subclinical abnormalities) after 6 months of dietary restriction, suggesting that cortical excitability in asymptomatic subjects with CD is modified by the adoption of the diet [20]. These findings support the hypothesis that the more prolonged is the dietary intervention, the more likely the cortical changes detected in newly diagnosed patients may be recovered.

Accordingly, the aim of the present study was to explore the changes in intracortical excitability and cortico-spinal conductivity to TMS after a much longer period of GFD. The hypothesis is that a long-lasting adherence to an appropriate dietary regimen might restore the balance between intracortical excitatory and inhibitory circuits.

### Transcranial Magnetic Stimulation measures

#### Motor evoked potentials (MEPs)

A single TMS pulse applied to the primary motor cortex (M1) elicits a MEP in contralateral target muscles, thus providing a functional assessment of the cortico-spinal conduction [21]. In particular, the MEP latency and the central motor conduction time, defined as the latency difference between the MEPs induced by M1 stimulation and those evoked by motor root stimulation, are indexes of integrity of the cortical-spinal pathways. The MEP amplitude reflects an aggregate measure of the excitation state of output cells in the motor cortex, nerve roots and conduction along the peripheral motor pathway to the muscles [6]. MEPs evoked by magnetic stimulation are produced by indirect activation of pyramidal cells, through cortico-cortical connections from the main source of inputs to the cortico-spinal cells represented by layer 2/3 and pyramidal neurons. Thus, the amplitude of MEPs reflects a balance of inhibitory and excitatory intracortical circuits activated by TMS, as well as the excitability of cortico-cortical projections to cortical-spinal neurons [22]. This is particularly true at higher stimulus intensities that produce a more prolonged activation of cortical networks resulting in a high frequency repetitive discharge of cortico-spinal cells [22].

#### Resting motor threshold (rMT)

The rMT provides information about a central core of neurons in the muscle representation of the M1 [23]. Resting MT is increased by drugs that block voltage-gated sodium channels [24] but it is not affected by drugs with effects on GABA [25]. Conversely, rMT is reduced by drugs increasing glutamatergic transmission not mediated by N-methyl-D-aspartate (NMDA) receptors [16], suggesting that rMT reflects both neuronal membrane excitability and non-NMDA receptor glutamatergic neurotransmission. MT is typically increased when a significant portion of the cortical-spinal tract is damaged, while it decreases in situations of hyperexcitable cortical-spinal system [5].

#### Cortical silent period (CSP)

When the single magnetic pulse is delivered during a voluntary contraction of the contralateral target muscle, the MEP is followed by a suppression of the electromyographic (EMG) activity [5]. This phenomenon, called CSP, is a measure of the suppression of the cortical-spinal output at a cortical level, probably due to the activation, after an early spinal phase (first 50–75 ms), of inhibitory cortical interneurons mainly mediated by GABA-B transmission [26, 27]. As known, interindividual differences and the inter-session variability of the CSP duration may be large, highlighting the importance of a standardized method of recording and analysis [6, 28, 29].

#### Short-latency intracortical inhibition (SICI) and intracortical facilitation (ICF)

Inhibitory and excitatory interneuronal activity within the human cortex can be studied non-invasively with paired-pulse TMS paradigm by using a “conditioning stimulus” (subthreshold) followed by a “test stimulus” (suprathreshold) [30, 31]. By varying the intensity of the conditioning stimulus and the interval between the pair of TMS pulses (interstimulus interval—ISI), a number of measures of intracortical interneuronal function and interaction have been developed. At ISI of 1–4 ms, the conditioning stimulus results in a reduction of MEP amplitude, and has been termed SICI; at longer ISI (7–20 ms), the effect is an ICF of the amplitude of motor responses [30, 31]. SICI is probably mediated by the activity of intracortical GABA-A interneurons [15, 16]. ICF is a more complex phenomenon as it is probably related to the activation a cortical circuit projecting upon cortico-spinal cells different from that preferentially activated by single pulse TMS, and probably composed of interneurons with less pronounced oscillatory properties. ICF seems to be dependent, to a great extent, on the activity of glutamatergic excitatory interneurons, although it is also modulated by other transmission pathways [16, 22].

## Materials and methods

### Ethics statement

The study was approved by the Ethics Committee of the “*Azienda Ospedaliero-Universitaria Policlinico-Vittorio Emanuele*”, Catania, Italy. All persons gave their written informed consent prior to their inclusion in the study, in accordance with the ethical standards laid down in the 1964 Declaration of Helsinki and its later amendments. All assessments were performed in a controlled laboratory environment by trained operators.

## Subjects

Twenty patients (mean age 35.10 ± 6.02 years; 6 males) with a diagnosis of CD according to the European Society for Paediatric Gastroenterology Hepatology and Nutrition (ESPGHAN) guidelines [32] were recruited from the Regional Center for Celiac Disease of the University of Catania (Italy). The mean age at the diagnosis was 27.25 ± 6.77 years, and they were enrolled after a mean time lag of 8.35 ± 2.74 years of strict GFD. Based on the international “ACG Clinical Guidelines for the diagnosis and management of Celiac Disease” [33], these patients underwent periodic visits with both CD specialist and skilled dietician, as well as structured survey to evaluate adherence to the diet [34], which resulted to be adequate in all of them. The clinical-electrophysiological data of these participants were compared with those obtained in twenty *de novo* patients not gluten restricted (mean age 35.00 ± 12.03 years; 4 males), recruited from the same Center and included in our previous study [17]. The clinical-serological features and the main findings from the diagnostic work-up of patients on GFD are summarized in Table 1, whereas those of newly diagnosed are described in the previous work.[17]. Twenty healthy volunteers (mean age 33.40 ± 8.20 years; 8 males) were included as a control group. Participants in each of the three groups were all right-handed.

**Table 1:** Clinical-serological features and diagnostic work-up of CD patients on gluten-free diet.

Patient	Age at onset	Symptoms at onset	Symptoms duration	Symptoms regression	GFD (years)	GFD adherence	Co-morbidities	Antibodies	tTG conversion	Histopathology
1	25	Weight loss, dyspepsia, diarrhoea, abdominal pain, anemia	More than 3 years	Within 2 years	7	3	-	tTG, EMA	Within 1 year	3c
2	24	Weight loss, dyspepsia, diarrhoea, abdominal pain, anemia	More than 1 year	Within 1 year	11	4	Hypothiroidism (on hormone replacement therapy)	tTG, EMA	Within 6 months	3b
3	30	Irritable bowel-like syndrome	More than 3 years	Within 2 years	8	4	Rosacea	tTG, EMA	Within 6 months	3c
4	29	Dyspepsia, diarrhoea, abdominal pain,	More than 3 years	Within 1 year	6	3	Dyslipidemia	tTG, EMA	Within 6 months	3c
5	17	Dyspepsia, diarrhoea, abdominal pain,	More than 1 year	Within 1 year	15	3	-	tTG, EMA	Within 6 months	3c
6	27	Dyspepsia, diarrhoea, abdominal pain,	More than 1 year	Within 1 year	10	3	-	tTG, EMA	Within 1 year	3c
7	35	Anemia	More than 1 year	More than 3 years	5	4	-	tTG, EMA	More than 1 year	3c
8	30	Elevated transaminases	More than 5 years	Within 1 year	9	3	-	tTG, EMA	More than 1 year	3a
9	31	Anemia	More than 1 year	Within 1 year	8	4	-	tTG, EMA	Within 6 months	3b
10	16	Weight loss, dyspepsia, diarrhoea, abdominal pain, anaemia	More than 1 year	Within 2 years	12	3	Hypothiroidism (on hormone replacement therapy)	tTG, EMA	Within 6 months	3c
11	21	None (screening, family history for CD)	-	-	8	3	Thyroiditis	tTG, EMA	More than 1 year	3c
12	30	Weight loss, dyspepsia, diarrhoea, abdominal pain	More than 5 years	Within 1 year	13	4	Rosacea	tTG, EMA	Within 6 months	3c
13	22	Anemia	More than 1 year	Within 2 years	5	4	-	tTG, EMA	Within 6 months	3c
14	21	Dyspepsia, diarrhoea, abdominal pain	More than 5 years	Within 1 year	8	4	Osteopenia	tTG, EMA	Within 6 months	3c
15	21	Dyspepsia, diarrhoea, abdominal pain	More than 1 year	Within 1 year	8	4	-	tTG, EMA	Within 6 months	3c
16	25	Anemia	More than 1 year	Still present	5	4	Lactose intolerance	tTG, EMA	Within 6 months	3b
17	42	None (screening, family history for CD)	-	-	6	4	-	tTG	Within 6 months	3c
18	35	Anemia	More than 3 years	Within 1 year	6	4	-	tTG, EMA	Within 6 months	3c
19	37	Anemia	More than 5 years	Still present	8	3	-	tTG, EMA	Within 6 months	3c
20	27	Weight loss, dyspepsia, diarrhoea, abdominal pain	More than 3 years	Within 2 years	9	3	-	tTG, EMA	Within 1 year	3b

CD = celiac disease; GFD = gluten-free diet; tTG = tissue transglutaminase antibodies; EMA = endomysial antibodies. Histopathological classification according to the Marsh-Oberhuber grading system [33]: 3a = mild villous flattening; 3b = marked villous flattening; 3c = total villous flattening. The adherence to the gluten-free diet (GFD) is based on a validated score [34]: patients with a score of 0 or 1 do not follow a GFD; patients with a score of 2 follow a GFD but with important errors that require correction; patients with a score of 3 or 4 follow a strict GFD.

All participants were matched for both age and educational level. Exclusion criteria were: major neurological disorder (i.e. Multiple Sclerosis, stroke, Parkinson’s disease, dementia, etc.); head trauma or epilepsy; acute, chronic or not compensated medical illness (i.e. myocardial infarction, kidney or liver failure, heart failure, etc.); age < 18 years; alcohol or drug abuse; use of drugs affecting cortical excitability (i.e. mood stabilizers, clonidine, benzodiazepines, antidepressants, antipsychotics, etc.); any condition precluding TMS execution.

### Assessment

The clinical-demographic evaluation included: age, gender, education, handedness, social and living conditions, general and neurological examinations, comorbidities. Neuropsychological tests included a screening of overall cognitive functions (Mini Mental State Examination), evaluation of neuropsychiatric symptoms (Neuropsychiatric Inventory) [35], a Structured Clinical Interview for DSM-IV Axis I Disorders, and the 17-items Hamilton Depression Rating Scale for the quantification of depressive symptoms [36]. Cognitive assessment was performed by a physician blind to the aim of the study.

Instrumental investigations included standard electroencephalogram (EEG), brain computed tomography (CT) scan and both single- and paired-pulse TMS. EEG was recorded by means of a Micromed Brain Quick (System Plus), with a standard montage according to the 10–20 International System and a pre-cabled EEG head cap. Brain CT was acquired with a helical 64-slices General Electric Scanning, with 2,5 mm slice thickness. Healthy controls underwent clinical, neuropsychological and TMS studies only.

### TMS procedure

TMS was performed using a High-power Magstim 200 magnetic stimulator (Magstim Co., Whitland, Dyfed, UK). A 70 mm figure-of-eight coil was held over the M1 at the optimum scalp position to elicit MEPs in the contralateral First Dorsal Interosseous (FDI) muscle of the dominant hand (the right hand in all participants), according to the Edinburgh Handedness Inventory [37]. EMG activity was recorded with silver/silver-chloride disposable self-adhesive and self-conductive surface electrodes. The active electrode was placed over the muscular belly of the target muscle, the reference distally at the metacarpal-phalange joint of the index finger, and the ground on the dorsal face of the wrist. For motor nerve conduction study, a bipolar nerve stimulation electrode with 6-mm diameter felt pads and an interelectrode separation of 25 mm was used.

The rMT was defined as the lowest stimulus intensity able to elicit MEPs at rest of an amplitude >50 μV in at least 5 of 10 trials [6]. MEP latency was calculated for each trial as the temporal interval from the TMS artifact to the first deflection of muscular response from the EMG baseline. As recommended, the MEP with the shortest cortical-motor latency was considered for each subject, since it is known to reflect the optimal conduction from M1 to the target muscle [6, 29]. Central motor conduction time was calculated by subtracting the conduction time in peripheral nerves, estimated by F wave techniques, from MEP latency obtained during moderate active muscle contraction, with a stimulus intensity set at 130% of the rMT. We identified the F waves according to the criteria reported by the International Federation of Clinical Neurophysiology (IFCN) as responses that are variable in their latency, amplitude, and configuration but that occurrence grouped with a consistent range of latency. The F wave with the shortest latency, providing a measure of conduction in the fastest motor axons [6], was considered. M wave (compound motor action potential—CMAP) and F wave were elicited by delivering supramaximal electrical stimulation to the ulnar nerve at wrist. M wave amplitude, F wave peak-to peak amplitude, and their minimum distal latencies were calculated; the latencies of M and F waves were visually assessed for each trial, although we considered the shortest latencies for both measures, as suggested by the latest IFCN guidelines on non-invasive electrical and magnetic stimulation [6]. Moreover, the F/M amplitude ratio, obtained for each subject, was defined as the mean amplitude of all F responses divided by the mean amplitude of the M wave. This ratio reflects the number and activation state of backfiring anterior horn cells, and therefore it is considered to be an index of spinal motor neuron excitability [38]. The size of the MEPs was expressed as a percentage of supramaximal M wave amplitude (MEP/CMAP x 100) (A ratio), which is known to reflect the central mechanisms contributing to the MEP amplitude. The use of A ratio also minimizes the inter-subject variability caused by inter-individual differences in peripheral MEP amplitude [6]. As recommended, in case of reduced amplitude, the TMS intensity has been gradually increased to test whether a higher stimulus intensity increases the A ratio [6]. The CSP was determined with an approximately 50% of maximum tonic voluntary contraction of the FDI muscle, induced by single TMS pulses delivered at 130% of rMT. Given that fatigue can modulate GABA-B-mediated intracortical inhibition [39], an intertrial interval >10 sec was used to allow subjects to relax between pulses; an adequate pause was also inserted whenever necessary to avoid muscle fatigue. Following the IFCN recommendations, the mean CSP duration based on trial-by-trial measurements of 10 rectified traces was calculated. In a single trial, the CSP was measured as the time elapsing from the onset of the MEP until the recurrence of voluntary tonic EMG activity. If voluntary EMG activity did not recover abruptly but gradually, making the identification of the end of the CSP difficult, the following criterion on a single trial basis was used: when the EMG activity reached or exceeded the pre-TMS baseline level and lasted for at least 50 ms, reoccurring EMG activity marked the end of the CSP [6, 29].

Paired-pulse TMS was obtained with a 70-mm figure-of-eight coil deriving pulses from a couple of Magstim 200 Stimulators, connected each other through a BiStim module (The Magstim Company, Whitland, Dyfed). The BiStim was connected to a CED Micro 1401 interface (Cambridge Electronic Design, Cambridge, UK) allowing stimulus generation and data capture. The conditioning stimulus was set at 80% of the subjects rMT, whereas the test stimulus at 130% of the rMT. The ISIs tested were 2 and 15 ms. Ten trials for each ISI were recorded in a random way with an 8-s interval between each trial. The amount of inhibition and facilitation was expressed as the peak-to peak amplitude ratio between the MEP amplitude produced by paired stimulation and that produced by test stimulus alone. Given that the amount of inhibition is influenced by the size of the test stimulus [40, 41], the MEP amplitude of the test stimulus alone at paired-pulse TMS was calculated in each group and compared.

All measurements were conducted while subjects were seated on a comfortable chair with continuous EMG monitoring to ensure either a constant level of activity during tonic contraction or complete relaxation at rest. As recommended, the rMT at the “hot spot” for the FDI muscle of each participant was re-assessed for each configuration (single-pulse TMS; paired-pulse TMS) [6]. In addition, trials that at visual inspection were contaminated with background EMG activity preceding the TMS pulse were excluded from the analysis. Data were collected and stored on a computer with an *ad hoc* software, allowing data acquisition, processing and analysis [42]. To minimize the inter-subject variability, all procedure was performed in the same laboratory by the same operators and time during the day.

### Statistical analysis

Because of the non-normal distribution of some variables, the differences between the several continuous variables obtained in the different groups of subjects (patients and controls) were evaluated by means of the non-parametric Kruskal-Wallis ANOVA, followed by the Mann-Whitney test for independent datasets, used as a *post hoc* test for the comparison of each pair of groups, when appropriate. For categorical variables, the Chi-square test was used. P value was considered statistically significant when <0.05.

## Results

Demographic and clinical characteristics of all participants are summarized in Table 2. The neurological and general examinations of the CD group was normal. As previously reported [17], nine *de novo* patients had autoimmune comorbidities, the most common being positive antithyroid peroxidase autoantibodies (six, although euthyroid), followed by asthma (two) and vitiligo (one). EEG and CT scan ruled out epileptic changes as well as intracranial calcifications or other clear neuroradiological abnormalities in all patients.

**Table 2:** Clinical-psychopathological features of subjects included in the study.

	(1) Controls		(2) *De novo* CD patients		(3) CD patients on GFD		Kruskal-Wallis ANOVA		Mann-Whitney test *p* =		
	Median	IQ range	Median	IQ range	Median	IQ range	H_(2,60)_	*p* =	1 *vs*. 2	1 *vs*. 3	2 *vs*. 3
**Age, years**	29.50	26.00‒42.00	33.00	24.00-45.00	35.50	29.50-39.00	0.601	0.740	-	-	-
**Education, years**	14.00	13.50-16.00	13.00	13.00-13.00	15.50	13.00-18.00	3.380	0.501	-	-	-
**MMSE**	30.00	29.00-30.00	30.00	29.00-30.00	30.00	30.00-30.00	3.155	0.520	-	-	-
**HDRS**	2.00	0.00-4.00	7.00	2.00-9.00	2.00	0.50-5.00	9.272	**0.01**	**0.006**	0.849	**0.015**
**NPI**	0.00	0.00-1.00	4.50	2.00-12.00	0.50	0.00-2.00	19.338	**0.0001**	**0.00012**	0.379	**0.0009**
							**Chi-square**	*p =*			
**Sex, males/females**	8/12		4/16		6/14		1.90	0.386			
**Co-morbidity, yes/no**	0/20		9/11		8/12		12.0	**0.002**			
**SCID-I, yes/no**	0/20		5/15		0/20		10.9	**0.004**			

CD = celiac disease; GFD = gluten-free diet; IQ = interquartile range; MMSE = Mini Mental State Examination; HDRS = 17 item-Hamilton Depression Rating Scale; NPI = neuropsychiatric inventory; SCID-I = Structured Clinical Interview for DSM-IV Axis I; NS = not significant; bold numbers = statistically significant *p* values.

*De novo* subjects exhibited significantly worse scores at the Hamilton Depression Rating Scale scores compared to controls and GFD patients, whereas the Structured Clinical Interview for DSM-IV Axis I Disorders disclosed a dysthymic disorder in five of them. Similarly, scores at the Neuropsychiatric Inventory (items of depression, anxiety, and irritability) were higher in non-gluten-restricted patients with respect to the other two groups.

**Fig 1:**
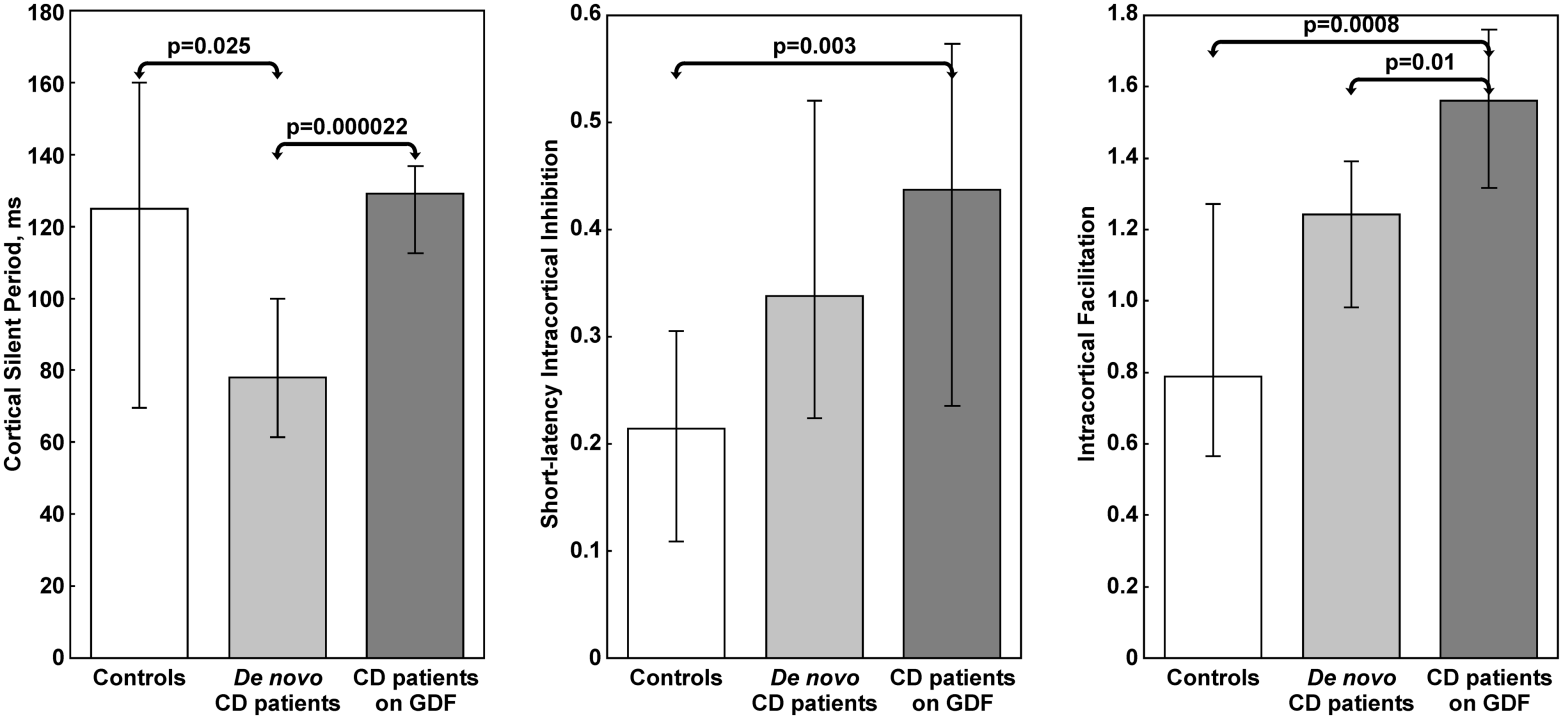
Comparison of cortical silent period, short-latency intracortical inhibition, and intracortical facilitation in patients and controls. Data are shown as median (columns) and interquartile range (whiskers). CD = Celiac disease; GFD = gluten-free diet.

Among the single-pulse TMS measures (Table 3 and Fig 1), the CSP was shorter in *de novo* subjects than in GFD patients (Kruskal-Wallis ANOVA, *p* = 0.000022) and controls (Kruskal-Wallis ANOVA, *p* = 0.025), without difference between these latter two groups. Conversely, the A ratio was significantly smaller in all patients than in controls, regardless of the dietary regimen. At paired-pulse TMS, notwithstanding the diet and compared to the other two groups, GFD patients exhibited a statistically significant decrease of the SICI (Kruskal-Wallis ANOVA, *p* = 0.003) and an enhancement of the ICF (Kruskal-Wallis ANOVA, *p* = 0.0008); conversely, SICI and ICF did not show relevant differences between *de novo* subjects and controls, as well as in the groups of patients before and after the diet, except for an increase of ICF in gluten-restricted patients compared to those non-restricted (Kruskal-Wallis ANOVA, *p* = 0.01).

**Table 3:** Comparison of electrophysiological data in patients and controls.

	(1) Controls		(2) *De novo* CD patients		(3) CD patients on GFD		Kruskal-Wallis ANOVA		Mann-Whitney test *p =*		
	Median	IQ range	Median	IQ range	Median	IQ range	H_(2,60)_	*p =*	1 *vs*. 2	1 *vs*. 3	2 *vs*. 3
**rMT, %**	37.00	32.00-40.00	35.00	34.00-41.50	38.00	36.00-40.50	0.890	0.713	-	-	-
**TS (130% rMT), mV**	0.66	0.45-0.91	0.78	0.46-0.96	0.68	0.45-0.76	1.614	0.649			
**MEP latency, ms**	19.25	18.45-20.20	18.60	18.20-19.65	18.90	18.45-19.30	1.179	0.689	-	-	-
**CMCT, ms**	5.80	5.55-6.55	5.90	4.45-6.30	5.20	4.65-5.80	2.425	0.582	-	-	-
**CMCT-F, ms**	5.30	4.15-5.65	4.80	4.45-5.55	4.38	3.68-4.90	2.413	0.585	-	-	-
**A ratio**	0.49	0.33-0.69	0.24	0.17-0.47	0.37	0.29-0.50	3.716	**0.0044**	**0.0025**	**0.04**	0.081
**M wave amplitude, mV**	12.93	11.50-15.35	12.51	10.81-15.16	11.58	8.58-14.52	0.931	0.710	-	-	-
**M wave latency, ms**	2.96	2.45-3.38	2.70	2.35-3.33	2.82	2.50-3.08	0.620	0.736	-	-	-
**F wave amplitude, μV**	0.11	0.07-0.15	0.10	0.06-0.16	0.13	0.07-0.17	0.206	0.772	-	-	-
**F wave latency, ms**	25.80	24.70-26.80	25.85	24.55-27.05	26.51	25.70-27.30	1.327	0.676	-	-	-
**F/M ratio**	0.01	0.01-0.01	0.01	0.01-0.01	0.01	0.01-0.02	0.523	0.775	-	-	-

CD = Celiac disease; GFD = gluten-free diet; IQ = intequartile; rMT = resting motor threshold; TS = amplitude of the motor evoked potential used as test stimulus at the paired-pulse TMS; MEP = motor evoked potential; CMCT = central motor conduction time; CMCT-F = central motor conduction time estimated by using the F-wave latency; A ratio = MEP/CMAP amplitude ratio; F/M ratio = F wave/CMAP amplitude ratio; NS = not significant; bold numbers = statistically significant *p* values.

**Fig 2:**
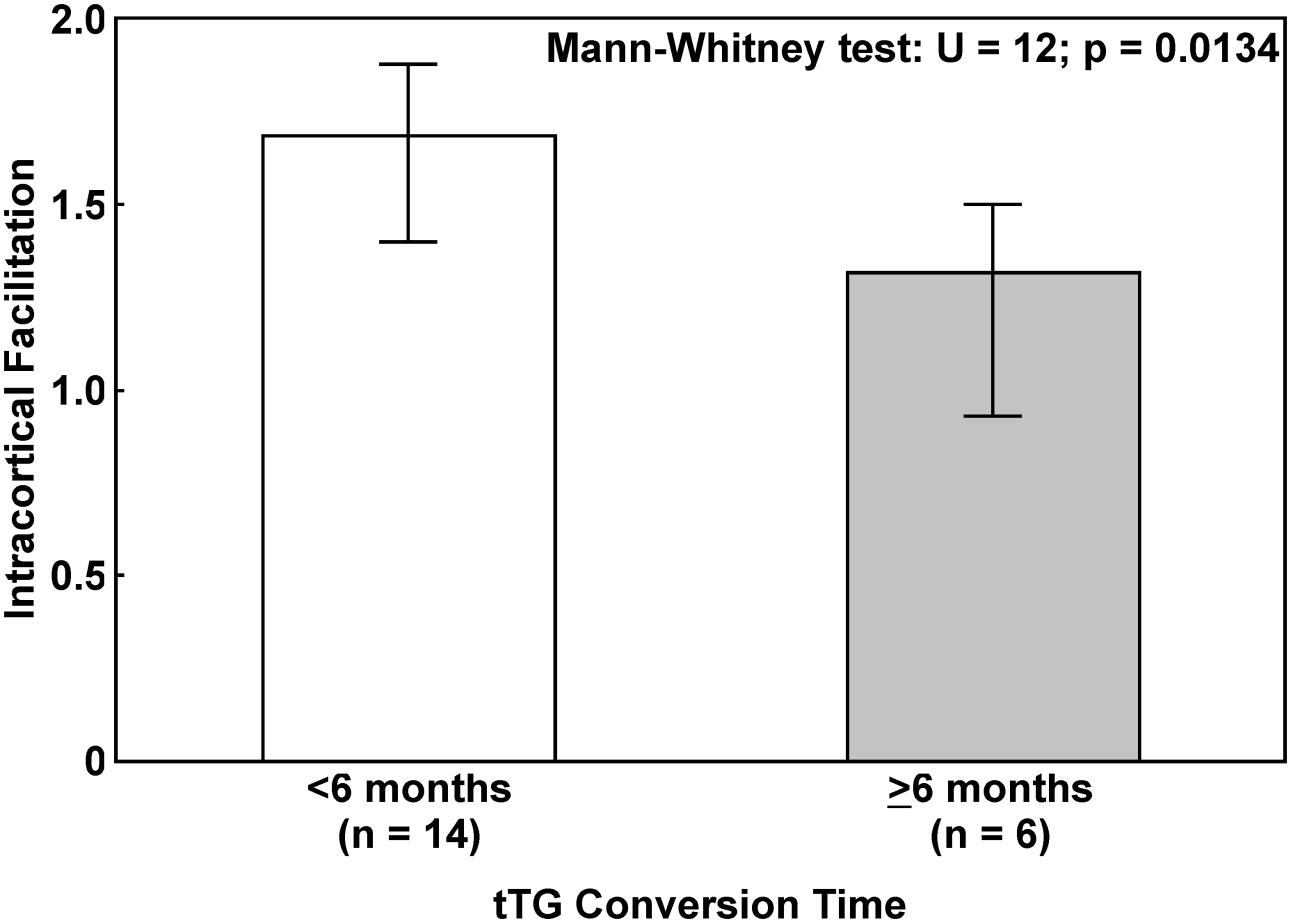
Comparison of intracortical facilitation in patients on gluten-free diet. Subjects are subdivided into two groups based on tissue transglutaminase antibodies (tTG) conversion time. Data are shown as median (columns) and interquartile range (whiskers).

Finally, the comparison of ICF in GFD patients subdivided into two groups based on their tissue transglutaminase (tTG) conversion time (Fig 2) showed a significant gradual decrease from those with a shorter conversion time (<6 months) to those with a longer conversion time (>6 months), whose ICF was more similar to that of *de novo* patients before the diet (Mann-Whitney test, *p* = 0.0134).

## Discussion

This is the first neurophysiological investigation examining the long-term impact of the GFD on motor cortex excitability and conductivity in adult celiac patients. The main finding is that a long-lasting appropriate GFD was effective in modulating the pattern of cortical excitability towards a normal level. Indeed, unlike the previous study where the length of dietary regimen was relatively short with the serum antibodies still present in a relevant proportion of subjects [18], here two of the measures of global excitation and inhibition of the motor cortex (namely rMT and CSP) were similar to those of healthy controls, suggesting a “restorative” role of a long-term GFD evaluated with TMS. However, data also indicate that there might be a differential involvement of specific cortical circuits in CD, with some changes responding to GFD and others that persist.

Single-pulse TMS showed there was a reduced A ratio at high intensity magnetic stimulation (130% rMT), in association with normal rMT. This indicates both a reduced excitability of excitatory cortico-cortical projections to cortico-spinal cells and of the balance of inhibitory and excitatory intracortical circuits activated by TMS. The results seem to converge consistently when considering paired-pulse TMS-derived measures. In particular, the reduction of intracortical inhibition as evaluated by SICI provides further evidence in favor of an abnormality of intracortical connections involving inhibitory GABA-A interneurons in newly diagnosed patients. It is worth noting that the extent of SICI was similar between *de novo* and GFD patients, suggesting that changes of intracortical inhibitory interneurons might be present in newly diagnosed subjects as well, and persist notwithstanding the diet. In GFD patients, especially in those with a shorter tTG conversion time, an enhancement of ICF might represent a compensatory phenomenon for a dysfunctional network within the intracortical interneurons that project into cortico-spinal cells. In other words, an intracortical synaptic dysfunction, mostly involving excitatory interneurons that reflects the activity of cortico-cortical connections different from those preferentially activated by single pulse stimulation [43] may occur in CD and poorly respond to the GFD.

This hypothesis is in accordance with the evidence of cortical, deep brain nuclei and white matter changes in CD, even without overt neurological symptoms. In particular, by using Magnetic Resonance Imaging automated volumetric analyses, a silent neurological involvement in biopsy-defined patients was demonstrated in terms of bilateral decrease of cortical gray matter and caudate nuclei volumes compared to controls, with a significant negative correlations between disease duration and volumes of the affected regions [44]. Similarly, voxel-based morphometry in biopsy-proven CD showed areas of significant gray matter loss, including medial perirolandic regions, dorsal frontal lobe and anterior cingulated cortex [45].

Because peripheral nerve and spinal cord involvement has been reported in patients with CD [46, 47], it can be speculated that the decreased A ratio in our patients could be related to a damage of the peripheral and/or cortico-spinal motor axons. However, the absence of clinical signs of neuropathy together with normal conduction velocity and peripheral nerve excitability rule out this scenario, pointing at a central motor pathway involvement. A spinal cord pathology is also unlikely because there was no clinical sign of dorsal columns or cortico-spinal tract impariment; rMT was also normal, confirming the absence of significant abnormalities in cortico-spinal connections. Interestingly, an abnormality in central motor circuits has been suggested by previous studies reporting an association between CD and motor neuron diseases [48–50]; moreover, some researchers found transglutaminase-6 antibodies in the serum of patients with amyotrophic lateral sclerosis [51]. However, a large population-based cohort study found no relationship between biopsy-proven CD and subsequent amyotrophic lateral sclerosis [52].

A pivotal facet of this study regards a possible explanation of how or why CD-related pathology would modulate the TMS measures of cortical function, albeit the paucity of previous studies on this aspect does not allow to draw firm conclusions. The most accepted hypothesis is that molecular mimicry between gliadin and some neuronal proteins could lead to a cross-reaction of anti-gliadin antibodies (Abs) with nervous system antigens [53, 54]. In particular, anti-gliadin Abs show immunoreactivity to synapsin I, a neuronal phosphoprotein involved in forming and maintaining the reserve pool of synaptic vesicles and in managing neurotransmitter release [55, 56]. In CD patients, anti-gliadin Abs might interact with synapsin I affecting the normal balance between excitatory and inhibitory neural circuits. Another intriguing hypothesis involves GABA, the main inhibitory neurotransmitter synthesized from glutamate by the glutamic acid decarboxylase (GAD). Since GABA and GAD are also synthesized by neurons of the enteric plexus [57], anti-GAD Abs may arise in CD patients and interfere with GABAergic synaptic transmission [54, 58, 59]. Additional data from the humoral autoimmunity to neuronal antigens showed diffuse T-lymphocytic infiltration within the perivascular cuffing, with inflammatory cells that could possibly damage the blood-brain barrier and expose the cerebral tissues to Abs [4], thus driving altered ion levels. Taken together, these findings may lead to a vicious circle resulting in an imbalance between inhibitory and facilitatory neuronal excitability that can be tested and monitored by TMS.

It is worth to remind that neurological deficits may even develop despite an adequate adherence to a GFD [60–62]. Accordingly, the persistence of TMS changes may also indicate a glutamate-mediated cortical rearrangement, probably triggered by the immune system dysregulation in CD and related to phenomena of long-term cortical plasticity. Alternatively, other factors might be invoked: i) patients could be not entirely compliant to GFD, and even minimal gluten contamination can cause a persistent immune response with related neurological involvement [63]; ii) a gliadine-mediated inflammatory attack of the motor neurons or axons may take place; iii) different mechanisms that are independent to the GFD may contribute. Regarding the latter hypothesis, a study using somatosensory evoked potentials and EEG in two celiac patients with cortical myoclonus showed that enhanced excitability of the sensory-motor cortex may also arise as a distant effect of cerebellar pathology [64].

Regarding the limitations, the selection of neurologically asymptomatic patients may be one of these, although, at the same time, it may be a strength because we screened the subclinical CNS involvement before and after a long period of GFD. Another limitation, as usual in TMS research, is the relatively small sample size; however, our groups were homogenous in terms of age, disease onset and length of gluten restriction. A critical aspect regards the fact that, given that the amount of SICI is strongly related to the intensity of the conditioning [65] and test stimuli [40, 41], the use of a range of intensities, in particular for the conditioning stimulus, is recommended when comparing patients to controls [6]. Thus, the use of a single conditioning stimulus intensity limited the present study; a further limitation is that the intensity of the conditioning stimulus was determined relative to rMT while it would be more appropriate to set the intensity relative to the active MT [65] (not evaluated in this study). Finally, a cerebellar influence cannot be excluded, although we did not find clinical or CT evidence of cerebellar pathology in our patients.

In conclusion, this new investigation shows that a long period of GFD is required to recover from electrophysiological abnormalities indicative of cerebral cortex involvement revealed by TMS in adult CD patients. However, notwithstanding the diet, some subclinical functional changes persist, although their clinical significance and the impact on the course of CD and its neurological complications need to be determined. Further studies will contribute to better elucidate the neurophysiological involvement and the effects of the GFD on the “celiac brain”.
